# Outcomes of Older Patients (≥60 years) with New-Onset Idiopathic Nephrotic Syndrome Receiving Immunosuppressive Regimen: A Multicentre Study of 116 Patients

**DOI:** 10.3390/jcm8030298

**Published:** 2019-03-02

**Authors:** Eloïse Colliou, Alexandre Karras, Jean-Jacques Boffa, David Ribes, Cyril Garrouste, Moglie Le Quintrec, Eric Daugas, Antoine Huart, Didier Ducloux, Aurélie Hummel, Inès Ferrandiz, Nathalie Demoulin, Noémie Jourde-Chiche, Dominique Chauveau, Vincent Audard, Stanislas Faguer

**Affiliations:** 1 Département de Néphrologie et Transplantation d’Organes, Centre de Référence des Maladies Rénales Rares, Centre Hospitalier Universitaire de Toulouse, 31000 Toulouse, France; eloise.colliou@orange.fr (E.C.); ribes.d@chu-toulouse.fr (D.R.); huart.a@chu-toulouse.Fr (A.H.); ines.ferrandiz01@gmail.com (I.F.); chauveau.d@chu-toulouse.fr (D.C.); 2 Service de Néphrologie, Hôpital Européen-Georges Pompidou, Assistance Publique des Hôpitaux de Paris, 75015 Paris, France; Alexandre.karras@aphp.fr; 3 Service de Néphrologie, Hôpital Tenon, Assistance Publique des Hôpitaux de Paris, 75020 Paris, France; jean-jacques.boffa@aphp.fr; 4 Service de Néphrologie et Transplantation Rénale, Centre Hospitalier Universitaire de Clermont-Ferrand, 63000 Clermont-Ferrand, France; cgarrouste@chu-clermont-ferrand.fr; 5 Service de Néphrologie et Transplantation Rénale, Centre Hospitalier Universitaire de Montpellier, 34000 Montpellier, France; m-lequintrec-donnette@chu-montpellier.Fr; 6 Service de Néphrologie et Transplantation Rénale, Hôpital Bichat, Assistance Publique des Hôpitaux de Paris, 75018 Paris, France; eric.daugas@aphp.fr; 7 Service de Néphrologie et Transplantation Rénale, Centre Hospitalier Universitaire de Besançon, 25000 Besançon, France; dducloux@chu-besancon.fr; 8 Service de Néphrologie-Dialyse, Hôpital Necker, Assistance Publique des Hôpitaux de Paris, 75015 Paris, France; aurelie.hummel@aphp.fr; 9 Département de Néphrologie, Cliniques Universitaires Saint-Luc, Université Catholique de Louvain, 1348 Louvain, Belgique; nathalie.demoulain@uclouvain.be; 10 Aix-Marseille Université, C2VN, INSERM 1263, INRA 1260, Marseille 13005, France; noemie.jourde@ap-hm.fr; 11 Assistance Publique-Hopitaux de Marseille (AP-HM), Centre de Néphrologie et Transplantation rénale, 13005 Marseille, France; 12 Institut National de la Santé et de la Recherche Médicale, U1048 (Institut des Maladies Cardiovasculaires et Métaboliques-équipe 12), 31000 Toulouse, France; 13 Service de Néphrologie et Transplantation, Centre de Référence Maladie Rare Syndrome Néphrotique Idiopathique, Hôpital Mondor, Assistance Publique des Hôpitaux de Paris, Université Paris-Est Créteil (UPEC), INSERM U955, équipe 21, 94000 Créteil, France; vincent.audard@aphp.fr

**Keywords:** minimal change disease, focal segmental glomerulosclerosis, older patients, rituximab, infection

## Abstract

Because of its rarity, renal presentation and outcomes of idiopathic nephrotic syndrome (INS; minimal changes disease or focal and segmental glomerulosclerosis) has poorly been described in elderly patients, precluding an individualized therapy procedure. Whether immunosuppressive regimens formerly designed in children and young adults are safe and efficient in elderly remains elusive. In a large multicentric retrospective study that included 116 patients with INS and onset ≥ 60 years of age, we showed that cumulative incidence of renal response was 95% after frontline therapy, with an age-dependent median time-to-response (60 days before 70 years of age at the onset vs. 120 days after; *p* = 0.03). Cumulative incidence of relapse was 90% at 7 years, with relapse occurring continuously over time. After a median follow-up of 34 months (IQR (12; 57)), 7 patients had died (6%) and 5 reached end-stage renal disease. Complications were highly prevalent: diabetes mellitus (23.3%), hypertension (24.1%), infection requiring hospitalization (21.6%) and acute kidney injury (9.5%). Thus, in older patients with INS and receiving steroids, renal response is delayed and relapse is the rule. Alternative immunosuppressive regimens, including B-cells depleting agents as frontline therapy, should be tested in this subset of patients to improve the mid- to long-term outcomes.

## 1. Introduction

Idiopathic nephrotic syndrome (INS) is a rare renal disease characterized by a massive urinary loss of albumin culminating in nephrotic syndrome. Renal pathology shows a normal appearance of glomeruli by light microscopy (minimal changes disease (MCD)), negative immunofluorescence and foot-process fusion by electron microscopy. In a subset of patients, focal and segmental glomerulosclerosis (FSGS) develops, sometimes accompanied by mild to moderate deposition of IgM and C3 complement component within the mesangium [1]. FSGS scarring lesions can be identified at the onset or in advanced disease. Until the identification of anti-PLA2R antibodies, membranous nephropathy was included in the INS but is now considered as a different entity. In this manuscript, INS thus refers to MCD and idiopathic FSGS but excludes membranous nephropathy.

Molecular mechanisms that initiate INS remain elusive but acquired cytoskeleton disorganization in podocytes with subsequent dysfunction of the slit diaphragm is the hallmark of the disease [2,3,4]. Compelling evidences suggest that INS may be an immunological disorder [5], including the response obtained with many oral or intravenous immunosuppressive agents [1] and the recent detection of specific autoantibodies in a subset of children with relapsing INS [6].

According to paediatric recommendations developed by international renal societies [7,8,9], oral steroids with slow tapering over 6 to 12 months are given as the main frontline treatment of INS in adult patients. In adults, relapse and dependency to steroids are observed in up to 50% of patients and prompted the testing of alternative steroids-sparring immunosuppressive agents. Calcineurin inhibitors (CNI), especially ciclosporin-A (CsA), are mostly used as the second-line steroid-sparing drug and lead to complete response in up to 80% of patients with relapse in 30% to 55% [10,11]. Conflicting data also suggest that cyclophosphamide, mycophenolate mofetil and levamisole may help to spare steroids but their efficacy may be disappointing and adverse events are frequent [12]. More recently, the anti-CD20 monoclonal antibody rituximab (Roche, Basel, Switzerland) emerged as a promising therapy in both children and adult patients with steroid-dependent or frequently relapsing INS [13,14,15,16]. According to the definition, a complete response was observed in 90% of patients and rituximab significantly reduced the annual rate of relapse in steroids-dependent patients. To date, rituximab is mostly used as a second- or third-line treatment but Kim et al. very recently showed that B cell-derived interleukin-4 is a pivotal cytokine that can stimulate membrane ruffling of podocytes and detachment of podocytes from the basement membrane [17], thus questioning the use of anti-B cells therapy sooner in the course of the disease to prevent steroids- and CNI-related adverse events.

In adulthood, the presentation and natural history of INS, including response to treatment and rate of relapse have been far less described than in children. Management of older patients is also stressed by the frequent comorbidities (cardiovascular disease, diabetes mellitus, obesity) preceding INS and a higher risk of new-onset diabetes mellitus, hypertension, AKI or cancer while receiving steroids, CNI or alkylating agents. Alternative regimens to first-line oral steroid therapy have recently been proposed [18,19] but there is an unmet need to define the optimal immunosuppressive regimen in adult patients, especially in older patients (≥60 years old at the onset).

The aim of our study was to describe the characteristics at the onset and the natural history of INS in a large cohort of 116 older patients (≥60 years), to identify the predictive factors of renal response and to assess the risk of treatment-related adverse events when immunosuppressive regimens approved in children and young adults are used in older patients.

## 2. Material and Methods

### 2.1. Inclusion and Exclusion Criteria

This study retrospectively included elderly patients referred between 1 January 1988 and 3 March 2017 for idiopathic nephrotic syndrome to 11 renal units located in University teaching hospitals in France (Toulouse, Marseille, Clermont-Ferrand, Montpellier, Besancon, Paris, Créteil) and Belgium (Louvain). All patients fulfilled the following criteria: (1) Overt nephrotic syndrome at presentation (i.e., serum albumin < 30 g/L and proteinuria > 3 g/day or urinary protein to creatinine ratio (uPCr) > 3 g/g) with available renal pathology (including immunostaining), (2) Renal pathology suggestive of MCD or FSGS, without immune deposits except for mild to moderate polyclonal IgM and C3 component deposits, (3) An age at the onset of the nephrotic syndrome over or equal to 60 years. Patients with diabetes mellitus, mild to moderate diabetic nephropathy and sudden nephrotic syndrome were also included if steroids sensitivity was subsequently confirmed. Patients with concomitant B-cell haematological malignancy, recent intake of non-steroidal anti-inflammatory drugs or hepatitis B or C or HIV infection were excluded from the study. The study was conducted according the Declaration of Helsinki, revised in 2004. According to the French law on retrospective observational studies and the recommendations of our Institutional Review Board, the written informed consent requirement was waived. 

### 2.2. Data Collection and Definitions

Renal presentation (blood pressure, urinalysis, proteinuria, serum creatinine, estimated glomerular filtration rate using the CKD-EPI formula), response to treatment and adverse events (infections requiring hospitalization, cardiovascular events) were reviewed. Complete response was defined as a serum albumin > 30 g/L and uPCr < 0.3 g/g [1]. Partial response was defined as a serum albumin > 30 g/L and uPCr < 3.5 g/g and decreased by 50% as compared to baseline. Relapse was defined by the development of a nephrotic syndrome or by an uPCr > 1 g/g without an alternative cause of proteinuria and lasting at least 7 days [1]. Acute kidney injury was defined according to the KDIGO classification [20].

### 2.3. Statistical Analysis

Continuous variables were given as median and interquartile range (IQR), while categorical variables were given as number and percentage. Univariate (unadjusted) analysis was performed using the Mann-Whitney U-test for continuous variables and the Fischer exact test for categorical variables. Adjusted odds ratios were estimated by multivariable (step-by-step descending) logistic regression. 

Comparisons between cumulative incidences were performed using the Gray test. To estimate the cumulative incidence of renal response, time to response was defined as the time to response after the frontline therapy or the time from the onset to the introduction of an alternative therapy in patients receiving a second-line therapy due to the lack of complete response. Survival curves were plotted according to the Kaplan-Meier method and comparisons between groups were performed using the Log Rank test (univariate analysis). To be considered significant, the *p*-value had to be lower than 0.05. Statistical analyses were performed using Xlstat software (Addinsoft, Paris, France).

## 3. Results

**Figure 1:**
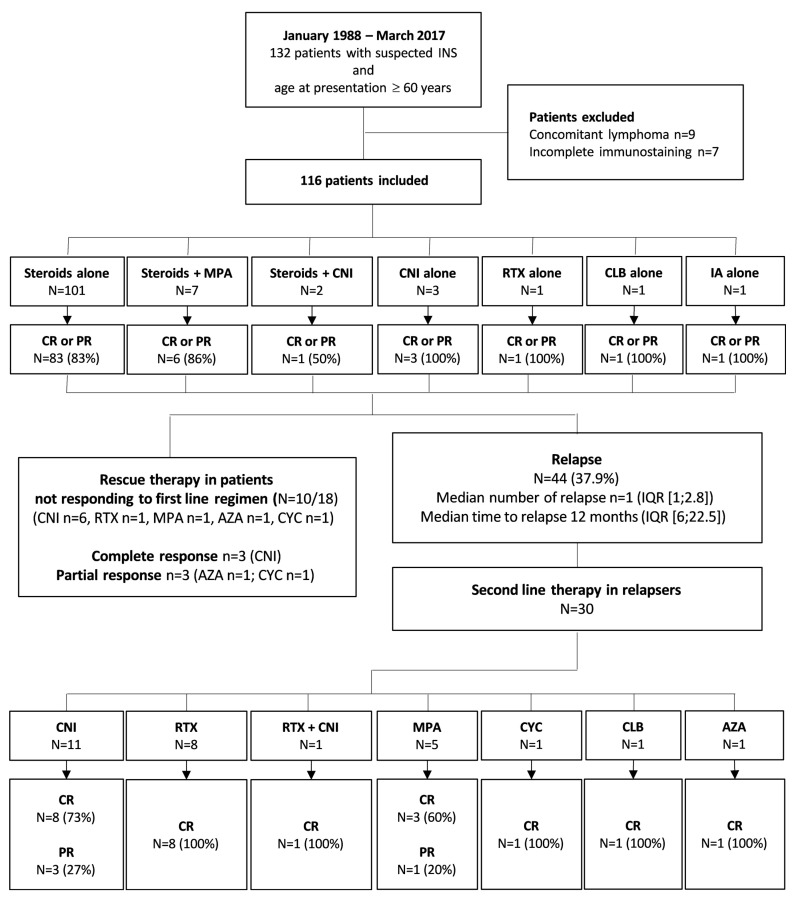
Flow Chart and Renal Outcomes of the 116 Patients Included in the Study.

116 patients were included in the study (median age at diagnosis: 68 years, IQR (64–77); male gender *n* = 72 (62%)) (Table 1 and Figure 1). Before the onset of the nephrotic syndrome, 19 patients (16.3%) had diabetes mellitus and 14 (12%) had another autoimmune disease (Supplementary Table S1). A diagnosis of solid cancer was made in 17 patients (14.7%) before (*n* = 14) or in the 6-months period (*n* = 3) following the onset of the nephrotic syndrome (median time between the diagnosis of cancer and nephrotic syndrome: 60 months (IQR (19–99)). Cancer was considered as active in 4 patients (pleural mesothelioma, melanoma, ovarian and prostate cancers) but none received tyrosine-kinase inhibitors or other drugs associated with secondary nephrotic syndrome. Treatment of the INS was not different in these patients.

### 3.1. Renal Presentation

At admission, median uPCr and serum albumin were 7 g/g (5–10.8) and 18 g/L (12–24.5), respectively. Median serum creatinine and estimated glomerular filtration rate (eGFR) were 121 μmol/L (89–200) and 50 mL/min/1.73 m^2^ (26–67), respectively. Sixty-one patients had criteria of acute kidney injury (AKI) according to the KDIGO classification (stage 1 *n* = 29; stage 2 *n* = 14; stage 3 *n* = 18; renal replacement therapy *n* = 2). Microscopic haematuria was identified in 51/114 individuals (44.7%) and *de novo* hypertension in 82/115 patients (71.3%).

Renal biopsy showed MCD and FSGS in 80 (69%) and 36 patients (31%), respectively. Mild and focal mesangial staining of polyclonal IgM and C3 in glomerulosclerosis area was identified in 23 (19.8%) and 32 (27.6%) cases. Features of acute tubular necrosis (tubular dilatation, loss of the brush border of proximal tubule cells, tubular cells shedding in the lumen) were observed in 42 patients (36.2%). Median percentages of sclerosed glomeruli and interstitial fibrosis were 5%, IQR (0–20) and 10%, IQR (0–15), respectively. Interstitial area was unremarkable otherwise. Superimposed atherosclerosis was identified in 7 individuals. No patients had features of diabetic nephropathy. 

### 3.2. Treatments of First Flare and Renal Outcomes

Frontline immunosuppressive regimens included steroids alone (*n* = 101, 87%; high doses 0.75 to 1 mg/kg/day) or in combination with mycophenolic acid (*n* = 7, 4.3%; low doses steroids 0.5 mg/kg/day *n* = 5/7) or a calcineurin inhibitor (*n* = 2, 1.7%), a calcineurin inhibitor alone (*n* = 3, 2.6%), rituximab alone (*n* = 1, 0.9%), immunoadsorption alone (*n* = 1, 0.9%) or chlorambucil alone (*n* = 1, 0.9%). Minimal dose of steroids was 1 mg/kg/day in all except 3 patients (0.75 mg/kg/day *n* = 2, 0.5 mg/kg/day *n* = 1) (Table 2). In addition, 78 patients (67.2%) received a renin-angiotensin-aldosterone system blocking agent. 

**Figure 2:**
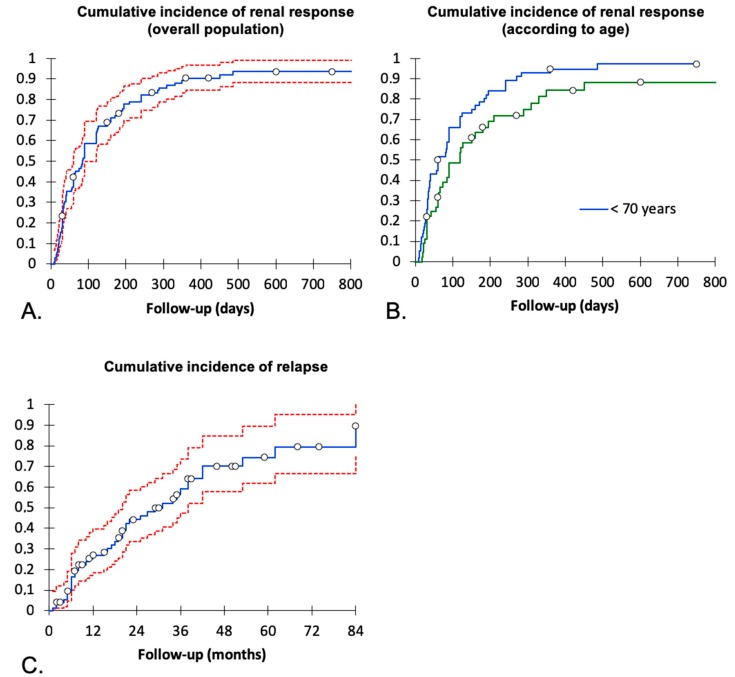
Outcomes of the idiopathic nephrotic syndrome in patients older than 60 years of age. Cumulative incidence of renal response (complete or partial) following the frontline therapy ((**A**) overall population. (**B**) Cumulative incidence according to the age at presentation, green line: age at the onset <70 years, blue line: age at the onset ≥70 years). Time of follow-up consisted of the time from presentation to the best renal response after the frontline therapy. In patients receiving a second- line therapy due to no or partial renal response, follow-up was stopped at the time of its introduction. In patients not responding and that did not receive a second-line therapy, last follow-up was used to estimate the cumulative incidence. (**C**) Cumulative incidence of renal relapse in patients that reached partial or complete response. Red lines represent the 95% confidence interval.

Assessment of frontline treatment was available for 114 patients (98.2%) since two were followed for less than one month. After a median follow-up of 34 months, 76 (65.5%) and 20 patients (17.2%) fulfilled the criteria of complete or partial response following the frontline therapy, respectively, whereas 18 (15.5%) had no response. The best renal response following the frontline therapy was reached after a median delay of 81 days (IQR (30–175)). Probability of renal response was then assessed by estimating the cumulative incidence of complete or partial response after the frontline therapy only. Cumulative incidence of renal response (partial plus complete) was about 95% following frontline treatment with time-to-response ≥6 months in 25% of patients (Figure 2A). Time-to-response was longer in the 62 patients older than 70 years at the onset of treatment (120 days) compared to the 54 patients younger than 70 years (60 days, *p* = 0.03) (Figure 2B). Steroids could be withdrawn in 61 (52.6%) after a median delay of 8 months (IQR (6–10)). Of note, renal pathology at presentation (i.e., MCD or FSGS) and the use of a renin-angiotensin-aldosterone system blocking agent did not modify the delay to the best renal response (data not shown). Of note, the best renal response in patients with MCD was complete or partial response in 75% and 9% compared with 44% and 36% patients with FSGS, respectively (*p* = 0.0003).

Among the 18 patients not responding to frontline treatment, renal pathology showed FSGS lesions in 5 (28%). No secondary cause was identified in these patients. A second-line treatment was given to 10 out of these 18 individuals (CNI *n* = 6, rituximab *n* = 1, MMF *n* = 1, azathioprine *n* = 1, cyclophosphamide *n* = 1) and only 5 of them (50%) reached complete (*n* = 3) or partial response (*n* = 2).

In the 5 patients with a previous history of diabetes mellitus and FSGS-INS on renal biopsy, four had complete or partial response following immunosuppressive treatment (steroids *n* = 3; cyclosporine-A *n* = 2). All had microscopic haematuria or polyclonal IgM/C3 mesangial deposits in the glomerulosclerosis area, thus confirming the diagnosis of primitive FSGS in these patients.

### 3.3. Management of Relapses and Incomplete Renal Response

A renal relapse occurred in 44 (37.9%) of the 96 patients that reached partial or complete response (median time to relapse 12 months (IQR (6–22.5))). As shown on Figure 2C that reports the cumulative incidence of relapse in patients that previously reached complete of partial response, the yearly percentage of relapse steadily increased from month 6 to month 42. At relapse, 25 had overt nephrotic syndrome and 18 had non-nephrotic proteinuria (uPCr > 1 g/g) (unavailable data in 1). At the time of relapse, 23 patients (52.7%) were still receiving immunosuppressive drugs, including steroids in 22 patients (median dose 7.5 mg/day).

30 patients received a non-steroid-based second-line therapy due to relapsing nephrotic syndrome (median number of relapses before the second-line *n* = 1, IQR (1–3)). Treatment-associated A short course of steroids to rituximab alone in 8, rituximab plus tacrolimus in 1, CNI alone in 11 (ciclosporin-A *n* = 10, tacrolimus *n* = 1), mycophenolic acid in 5, cyclophosphamide in 3, chlorambucil in 1 or azathioprine in 1 (Figure 2).

Among the 30 patients that received a second-line therapy due to a relapsing nephrotic syndrome, complete response was obtained in 8/8 (100%) patients receiving rituximab, 8/11 (73%) receiving CNI, 1/1 (100%) receiving rituximab + CNI, 3/5 (60%) receiving mycophenolic acid and steroids combination, 3/3 (100%) receiving cyclophosphamide, 1/1 receiving chlorambucil (100%) and 1/1 (100%) receiving azathioprine. Steroids were subsequently withdrawn in 23 patients (56%).

Overall, rituximab was used in 23 patients (Table 3), between frontline up to fourth-line therapy, alone (*n* = 1) or in combination with steroids (*n* = 21) or CNI (*n* = 1). Among these 23 patients, 14 (61%) and 4 (17%) reached complete or partial response. Of note, 8/18 patients with renal response following rituximab had features of idiopathic FSGS on renal biopsy and 16 had steroid sensitivity (complete response *n* = 12). In contrast, among the 5 patients that did not respond to rituximab, 2 did not respond to steroids and 3 had only partial response. Steroids were withdrawn in 15/21 after a median time of 7.2 months. Renal relapse occurred in 7 patients after a median time of 13 months [10,11,12,13,14,15,16,17,18,19,20,21,22,23] following rituximab infusion. In the four patients with available data, CD19^+^ B-cells were detectable in the blood at the time of the relapse.

### 3.4. Adverse Events and Long-Term Outcomes

After a median follow-up of 34 months (IQR (11.8–56.5), 7 patients had died (6%). The cause of death was cancer in 1 patient (with active melanoma at diagnosis), infectious disease in 3 (Supplementary Table S2), haemorrhagic shock in 1 and was not available in 2. Overall, 78 patients (67.2%) reached complete renal response and 30 (25.9%) had two or more relapses of the disease. In survivors, median eGFR at last follow-up was 64 mL/min/1.73 m^2^. Five patients reached end-stage renal disease (median delay 25 months) but none received renal transplantation. Four out of these five patients had stage 3 AKI at the diagnosis.

During follow-up, three patients developed deep venous thrombosis, including pulmonary embolism in one. None developed renal vein thrombosis. Eleven patients (9.5%) developed delayed AKI, including 8 that received CNI at the time of AKI. During the follow-up (i.e., at distance from the onset of INS), new-onset or aggravated diabetes and hypertension occurred in 27 (23.3%) and 28 (24.1%) patients. Twenty-five patients (21.6%) developed a severe infection requiring hospitalization, including 3 (12%) that led to death (Table 4 and Supplementary Table S2).

Other treatment or disease-related complications included osteoporosis, dyslipidaemia, psychiatric disorder, tremor, hirsutism and gingival hypertrophy in 14 (12.1%), 50 (43.1%), 19 (16.4%), 3 (2.6%), 4 (3.4%) and 5 (4.3%) patients, respectively. Seven patients (6.3%) developed peripheral adrenal failure requiring transient or long-lasting supplementation by hydrocortisone.

Lastly, fifteen patients (12.9%) developed a solid cancer after a median time of 23 months (IQR (13–44)) after the onset of the nephrotic syndrome (Supplementary Table S3). Age at the onset between patients that did or did not develop solid cancer was similar.

## 4. Discussion

Idiopathic nephrotic syndrome is a complex glomerular disorder with heterogeneous presentation, response to treatment and risk of relapse that vary according to the age of onset and underlying glomerular lesions (MCD vs. FSGS) [1]. MCD is the main cause of nephrotic syndrome in children while it accounts for only 10%–15% of nephrotic syndrome in adulthood. Epidemiological and descriptive studies are scarce in adulthood or included mainly individuals less than 60 years of age [18,19,21,22,23,24,25]. Thus, incidence and outcomes of INS in older patients remains unknown, whereas these patients are at higher risk of treatment-related complications.

In this series, we excluded patients with secondary forms of nephrotic syndrome (i.e., hepatitis, HIV, haematological, malignancies or drugs). However, our series underlined that some INSs develop in individuals with chronic unrelated systemic or localized disease (for instance, diabetes mellitus, thyroiditis and solid cancer). Therefore, the risk of therapy-related complications is probably higher than in younger adults.

To date, the only study that characterized the clinical presentation and outcomes of older patients with INS included only 15 patients [22]. Here, we reported the largest study of patients with INS that developed after 60 years (*n* = 116) and identify several age-dependent characteristics. We showed that 85% of patients that develop INS after the age of 60 have complete or partial renal response when receiving immunosuppressive regimens used in children. However, time to the best renal response was longer than younger individuals and 10 to 25% of patients developed severe infection, cardiovascular or metabolic complications, AKI or cancer due to the side effects of immunosuppression.

In adulthood, secondary causes of minimal change disease must be excluded, including malignancies, drugs, infections and autoimmune disorders. In our series, we excluded patients with haematological malignancies, as well as drug- and infection-related nephrotic syndrome. Patients with a history of solid cancer were included because a definite pathophysiological relationship could not be demonstrated and management of the patients was not modified according to the underlying cancer. Also, the association of autoimmune disorder and INS did not modify the immunosuppressive management of patients. Lastly, alternative pathological diagnosis should always be ruled out (including amyloidosis or C3 glomerulonephritis). To minimize the risk of inclusion biases despite the lack of electron microscopy, we excluded patients with incomplete renal immunostaining or with glomerular C3 deposits outside glomerulosclerosis lesions.

In our series that included older patients (>60 years of age), intense nephrotic syndrome with heavy proteinuria (median uPCR, 7 g/g) and severe hypoalbuminemia (median, 18 g/L) and frequent AKI were the hallmarks of renal presentation. Microhematuria was frequent at presentation (45% versus 20% in children), as well as hypertension (72%). In children and young adults, AKI may be observed in up to 40% of patients at the onset of INS [1,25]. Causes of INS-associated AKI was recently reviewed [26] and old age, severe hypoalbuminemia, a background of hypertension and vascular lesions on kidney biopsy emerged as risk factors. Indeed, using the standardized KDIGO categorization, we identified AKI in as much as 52% of older patients with INS. Moreover, 15% had severe KDIGO stage 3 AKI (15%) at presentation and 8% developed AKI during the follow-up. The high risk of AKI in this subset of patients should prompt the modulation of treatment according to this distinctive feature.

Hypercoagulability and an increased risk of venous thrombosis have been emphasized in patients with severe nephrotic syndrome [27]. However, only 3 patients (2.5%) developed venous thrombosis in our series compared to 11/125 (9%) patients in a large series that included young adults [25]. Due to the retrospective nature of the study, we could not review the anticoagulation treatments received by the patients at the onset and during follow-up. Therefore, the risk of thrombosis in old patients with INS could not be adequately assessed.

Almost 90% of our patients received high-doses steroids as an induction immunosuppressive regimen. In previously published cohorts (see review by Vivarelli et al. [1]) reporting children and adult patients with INS, the time-to-response to prednisone was longer in adulthood compared to childhood (median time 10 days in children versus 50 days in adult patients). In our series dedicated to older patients, the median time-to-response to prednisone was 81 days, suggesting that adult patients, especially older patients, have differences in pathophysiological mechanisms, immune response to steroids, superimposed age or hypertension-related chronic kidney disease or glomerulosclerosis; that may all promote persistent proteinuria. Interestingly, in recent studies from Li et al. and Remy et al. that included patients with MCD and with a mean age of 29 and 44 years at the onset, the rate of complete response were 70 and 60% at week 4, confirming our results suggesting an age-dependent time-to-response pattern among older patients in our series (60 days vs. 120 days in patients ≤ or ≥70 years of age) [18,19].

The cumulative incidence of renal response following the frontline therapy (i.e., related to the immunosuppressive treatment use at diagnosis or the natural course of the disease) was close to 95%. This finding confirms the theoretical need to extend the period of induction regimen up to 16 weeks prior to consider the INS steroid-resistant. However, we also showed that certain rare patients will spontaneously reach renal response one year beyond the frontline treatment. Nevertheless, older patients are at very high risk of complications (10% to 30% in our series), which are frequently related to the treatment (diabetes mellitus, hypertension, AKI, infection, cancer), suggesting that specific emphasis should be made on this topic. In particular, approximately one fifth of patients developed a severe infection that required hospitalization, including 3 that died from the infection. This unexpected high-rate of mortality was also observed in the control group of the MSN trial [18]. Diabetes mellitus, hypertension and AKI, three complications mainly related to the extended use of steroids and CNI, were also prevalent in our series. Thus, the use of alternative immunosuppressive schemes should be considered and prospectively tested in older patients to improve the overall outcome. However, tacrolimus and mycophenolate mofetil failed to show tolerance superiority over high-doses steroids [18,19].

The monoclonal anti-CD20 antibody rituximab (Roche, Basel, Switzerland) and more recently the anti-CD20 antibody ofatumumab (Novartis Europharm, UK), have emerged powerful and well tolerated treatments in both children and adults with MCD [28,29]. Rituximab reduces steroid- and CNI-dependency in patients with MCD sensitivity to these drugs and may be successful in certain patients with steroid-resistant INS [30]. A further comparative study in patients with steroid-resistant INS failed to extend these results [31]. In our series, rituximab was secondarily used in 20% of patients and led to renal response in 75% of them. Complete response was obtained in 10/11 patients (91%) receiving rituximab in first- or second-line. Thus, adding rituximab to a short course of steroids in frontline immunosuppressive regimens may help to control the nephrotic syndrome better and faster, leading to the reduced use of steroids and CNI and subsequent lowering the incidence of cardiovascular and metabolic complications, as well as AKI episodes. This approach was successfully tested in patients with membranous nephropathy [32]. To confirm this hypothesis, a prospective comparative study is mandatory.

Lastly, cumulative incidence of relapse was 25% at month 12 and 90% at month 84 in our series, without specific predictive factor. These rates were close to those reported in recent interventional studies [18,19]. Interestingly, relapses occurred continuously over time suggesting that permanent remission is not a common feature of INS in older patients. This should also prompt the testing of alternative immunosuppressive treatments to avoid long-term complications related to recurrent use of steroids or CNI, which are highly prevalent in this population.

Owing to its retrospective design, our study has several limitations. First, we included patients over a 30-yearperiod that may induce biases of recruitment and treatment heterogeneity. However, all patients except one received an immunosuppressive regimen including steroids as frontline therapy. Also, the lack of electron microscopy may have led to the inclusion of patients with other causes of glomerulopathy. To minimize this risk, we excluded patients without information regarding immune deposits or with underlying haematological malignancy. Lastly, follow-up was heterogeneous between patients but this study reports one of the larger cohorts of older patients with MCD or idiopathic FSGS, thus improving the knowledge of this rare glomerulopathy.

## 5. Conclusions

INS in patients older than 60 years of age is characterized by an age-dependent time-to-renal response and frequent cardiovascular, metabolic, infectious and renal complications. Renal response is obtained in most patients but relapse seems to be the rule. Alternative immunosuppressive regimens should be tested in a randomized study to improve the overall outcome of these patients.

## Figures and Tables

**Table 1:** Characteristics of Presentation of 116 Older Patients with Idiopathic Nephrotic Syndrome. IQR, interquartile range; INS, idiopathic nephrotic syndrome; eGFR, estimated glomerular filtration; MCD, minimal change disease; FSGS, focal and segmental glomerulosclerosis. * Cancer diagnosed up to 6 months after presentation was considered as previous or concomitant to the INS.

Characteristics	Patients *N* = 116
**Patients**	
Age at presentation (years; median (IQR))	68 (64–77)
Male gender (*n*, %)	72 (62)
BMI (kg/m²)	25.6 (23.4–28.3)
Diabetes mellitus (*n*, %)	19 (16.3)
Insulin (*n*, %)	11 (61.1)
Autoimmune disease (*n*, %)	14 (12)
Previous or concomitant cancer * (*n*, %)	17 (14.7)
Time between cancer and INS (months; median (IQR))	60 (19–99)
**Renal Presentation**	
UPCr (g/g; median (IQR))	7 (5–10.8)
Serum albumin (g/L; median (IQR))	18 (12–24.5)
Serum creatinine (µmol/L; median (IQR))	121 (89–200)
eGFR (ml/min/1.73 m^2^; median (IQR))	50 (26; 67)
Acute kidney injury (*n*, %)	61 (52.6)
Stage 1 (*n*, %)	29 (25)
Stage 2 (*n*, %)	14 (12.1)
Stage 3 (*n*, %)	18 (15.5)
Haematuria (*n*, %)	51 (44)
Hypertension (*n*, %)	82 (71)
**Renal Biopsy**	
MCD (*n*, %)	80 (69)
FSGS (*n*, %)	36 (31)
IgM deposits (*n*, %)	23 (19.8)
C3 deposits (*n*, %)	32 (27.6)
Acute tubular necrosis (*n*, %)	42 (36.2)
Percentage of glomerular sclerosis (median (IQR))	5 (0–20)
Percentage of interstitial fibrosis (median (IQR))	5 (0–15)

**Table 2:** Treatments and Outcomes. MMF, mycophenolate mofetil; CNI, calcineurin inhibitors; RAAS, renin-angiotensin-aldosterone system; IQR, interquartile ranges; eGFR, estimated glomerular filtration rate; ESRD, end-stage renal disease.

Characteristics	*N* = 116
**First-Line Treatment**	
Immunosuppressive regimen	
Steroids (*n*, %)	101 (87)
Pulses	5 (4.3)
MPA and steroids (*n*, %)	7 (6)
CNI (*n*, %)	3 (2.6)
CNI and steroids (*n*, %)	2 (1.7)
Rituximab (*n*, %)	1 (0.9)
Other treatments (*n*, %)	2 (1.7)
RAAS blocking agents (*n*, %)	78 (67.2)
**Response to Treatment**	
Complete response (*n*, %)	76 (65.5)
Partial response (*n*, %)	20 (17.2)
No response (*n*, %)	18 (15.5)
Time from the onset to the best renal response (days)	61 (30–123)
Withdrawal of steroids (*n*, %)	61 (52.6)
**Relapse (*n*, %)**	44 (37.9)
Time from the best renal response (months; median (IQR))	12 (6–22.5)
On-going immunosuppressive treatment at the relapse	23 (52.7)
**Status at Last Follow-Up**	
Follow-up duration (months; median (IQR))	34 (11.8–56.5)
Alive (*n*, %)	109 (94)
Sustained complete response (*n*, %)	78 (67.2)
eGFR (mL/min/1.73 m^2^)	64 (41–79)
ESRD (*n*, %)	5 (4.3)
Time interval from the onset (months; median (IQR))	25 (0–46)

**Table 3:** Outcomes of 23 older patients with INS receiving a monoclonal anti-CD20 antibody. CR, complete response; PR, partial response; NR, no response. * Relapse occurred after a median time of 13 months (10–23) following the use of rituximab.

Characteristics	*N* = 23	
**Line of treatment (n, %)**		
**First**	1 (4.3)	
**Second**	10 (43.6)	
**Third**	9 (39.1)	
**Fourth**	3 (13)	
**Delayed Neutropenia**	None	
**INS Response according to the Line of Treatment**		**Median Time to Response**
**First-Line, CR/PR/NR**	1/0/0	284 days
**Second-Line, CR/PR/NR**	9/0/1	42 days
**Third-Line, CR/PR/NR**	3/3/0	25 days
**Fourth-Line, CR/PR/NR**	1/0/0	30 days
Relapse after Rituximab (n, %) *		**Median Time to Response**
**First-Line**	1 (100)	15 months
**Second-Line**	2 (20)	33 months
**Third-Line**	3 (33)	10 months
**Fourth-Line**	1 (100)	11 months

**Table 4:** Main Adverse Events Occurring During the Follow-Up. *INS*, idiopathic nephrotic syndrome; *IQR*, interquartile ranges.

Adverse Events	*N* (%)
**Pulmonary Embolism**	1 (0.8)
**Deep Vein Thrombosis**	3 (2.9)
**Acute Kidney Injury**	11 (9.5)
**Diabetes Mellitus**	
***De novo***	16 (13.8)
**Worsening**	11 (9.5)
***De novo* Hypertension**	28 (24.1)
**Infection Requiring Hospitalization**	25 (21.6)
**Cancer**	15 (12.9)
**Time from the Onset of INS (months; median (IQR))**	23 (13–44)
**Osteoporosis**	14 (12.1)
**Dyslipidaemia**	50 (43.1)
**Psychiatric Disorder**	19 (16.4)
**Adrenal Failure**	7 (6.3)
**Significant Tremor**	3 (2.6)
**Hirsutism**	4 (3.4)
**Gingival Hypertrophy**	5 (4.3)

## Supplementary Materials

The following are available online at https://www.mdpi.com/2077-0383/8/3/298/s1, Table S1: Autoimmune diseases preceding the INS in 116 elderly, Table S2: Infections requiring hospitalization during the follow-up, Table S3: Cancers that developed more than 6 months after the onset of the INS.
