# Supplementary material for: Outcomes of Older Patients (≥60 years) with New-Onset Idiopathic Nephrotic Syndrome Receiving Immunosuppressive Regimen: A Multicentre Study of 116 Patients
Source: J Clin Med. 2019 Mar 2;8(3):298. doi: 10.3390/jcm8030298 (PMC6463053; doi:10.3390/jcm8030298)
Supplement: Supplementary file 1 [file jcm-08-00298-s001.pdf]

Supplementary Materials

Table S1. Autoimmune diseases preceding the INS in 116 elderly.

Autoimmune Diseases before SNI	Number
**Connective tissue diseases**	
Lupus	1
Antiphospholipid syndrome	1
Sjögren's disease	1
**Thyroid**	
Immune-related hypothyroidism	3
Graves' disease	2
**Intestinal/Liver**	
Colitis	
Hepatitis	1
Sclerosing cholangitis	1
**Others**	
Biermer's disease	1
Guillain-Barre syndrome	1
Graft versus host disease	1
Type 2 cryoglobulinemia	1

Table S2. Infections requiring hospitalization during the follow-up.

Infections	*N* = 15 Patients	Death (*n* = 3)
**Bacterial**		
Endocarditis	1	0
Arthritis	1	0
Prostatitis	1	0
Pneumoniae	5	0
Colitis	3	0
Bacteremia	5	1
Pyelonephritis	2	1
Soft tissue infection	7	0
Peritonitis	1	0
Ascites infection	1	0
**Viral**		
CMV proliferation	1	0
Influenza A	1	0
Zoster virus	2	0
**Fungal**		
Aspergillus sp.	1	1
Alternaria sp.	1	0

Table S3. Cancers that developed more than 6 months after the onset of the INS.

**Cancers**	***N* = 15**	**Time from the Onset of INS Median 32 Months** (18–52)
**Urological**		
Prostate	4	62
Kidney	1	34
Bladder	1	18
Urinary tract	1	7
**Skin**		
Epidermoid carcinoma	2	36
**Intestinal**		
Colon	1	12
Stromal tumor	1	47
Rectum	1	27
**B-cell proliferation**		
Macroglobulinemia	1	32
B-cell lymphoma	1	19
**Others**		
Breast	1	18
Epidermoid ENT	1	52
